# The Differential Effects of Propylthiouracil and Methimazole as Graves’ Disease Treatment on Vascular Atherosclerosis Markers: A Randomized Clinical Trial

**DOI:** 10.3389/fendo.2021.796194

**Published:** 2021-12-20

**Authors:** Wismandari Wisnu, Idrus Alwi, Nafrialdi Nafrialdi, Kuntjoro Harimurti, Tjokorda Gede D. Pemayun, Sri Widia A. Jusman, Dewi Irawati S. Santoso, Alida R. Harahap, Suhendro Suwarto, Imam Subekti

**Affiliations:** ^1^ Division of Endocrine, Metabolism and Diabetes, Department of Internal Medicine, Faculty of Medicine, Universitas Indonesia, Dr. Cipto Mangunkusumo General Hospital, Jakarta, Indonesia; ^2^ Division of Cardiology, Department of Internal Medicine, Faculty of Medicine, Universitas Indonesia, Dr. Cipto Mangunkusumo General Hospital, Jakarta, Indonesia; ^3^ Department of Pharmacology and Therapeutics, Faculty of Medicine, Universitas Indonesia, Jakarta, Indonesia; ^4^ Division of Geriatrics, Department of Internal Medicine, Faculty of Medicine, Universitas Indonesia, Dr. Cipto Mangunkusumo General Hospital, Jakarta, Indonesia; ^5^ Division of Endocrine, Metabolism, and Diabetes, Department of Internal Medicine, Faculty of Medicine, Diponegoro University, Dr. Kariadi General Hospital, Semarang, Indonesia; ^6^ Department of Biochemistry and Molecular Biology, Faculty of Medicine, Universitas Indonesia, Jakarta, Indonesia; ^7^ Department of Medical Physiology, Faculty of Medicine, Universitas Indonesia, Jakarta, Indonesia; ^8^ Eijkman Institute for Molecular Biology, Jakarta, Indonesia; ^9^ Division of Tropical and Infectious Disease, Department of Internal Medicine, Faculty of Medicine, Universitas Indonesia, Dr. Cipto Mangunkusumo General Hospital, Jakarta, Indonesia

**Keywords:** adhesion molecules, carotid intima media thickness, Graves’ disease, hyperthyroidism, methimazole, propylthiouracil, pulse wave velocity, vascular atherosclerosis

## Abstract

**Background:**

Hyperthyroidism is related to vascular atherosclerosis. Propylthiouracil (PTU) and methimazole, other than their antithyroid effects, may have different mechanisms in preventing atherogenesis in Graves’ disease.

**Objective:**

This study aimed to investigate the effect of antithyroid drugs on markers of vascular atherosclerosis in Graves’ hyperthyroidism.

**Methods:**

This study was a single-blind, randomized clinical trial conducted on 36 patients with Graves’ disease in Cipto Mangunkusumo General Hospital, Jakarta, Indonesia, from June 2019 until July 2020. Graves’ disease was diagnosed from clinical manifestation of hyperthyroidism with diffuse goiter and then confirmed by thyroid stimulation hormone (TSH), free T4 (fT4), and TSH-receptor antibody (TRAb) measurements. Participants were randomly assigned to either a PTU or a methimazole treatment group and followed up for 3 months. Markers of vascular atherosclerosis were represented by adhesion molecules [intercellular adhesion molecule-1 (ICAM-1), vascular cell adhesion molecule-1 (VCAM-1), and E-selectin], carotid artery stiffness [pulse wave velocity (PWV)], and thickness [carotid intima media thickness (cIMT)].

**Results:**

By the end of the study, 24 participants reached euthyroid condition (13 from the PTU group and 11 from the methimazole group). After 3 months of follow-up, in the PTU group, we noticed an improvement of ICAM-1 [pretreatment: 204.1 (61.3) vs. posttreatment: 141.6 (58.4) ng/ml; p = 0.001], VCAM-1 [837 (707–977) vs. 510 (402–630) ng/ml; p < 0.001] and E-selectin [32.1 (24.1–42.7) vs. 28.2 (21.6–36.8) ng/ml; p = 0.045] in the PTU group. In the methimazole group, only VCAM-1 improvement [725 (565–904) vs. 472 (367–590); p = 0.001] was observed. Meanwhile, we found no significant changes in PWV or cIMT in either group.

**Conclusion:**

Antithyroid treatment in Graves’ disease leads to improvement in adhesion molecules, with a lesser effect on methimazole, whereas there were no significant changes in PWV or cIMT. PTU may have a better mechanism compared with methimazole in terms of improving adhesion molecules.

## Introduction

Hyperthyroidism is a condition involving low thyroid-stimulating hormone (TSH) and high thyroid hormone levels in the body; Graves’ disease is the most frequent cause of this condition (1, 2). Hyperthyroidism leads to high levels of body metabolism, often inducing such complications as heart failure. Recent studies have found that hyperthyroidism has a potential correlation with atherosclerosis cardiovascular disease (ASCVD) (3–5). Free T4 (fT4) has been correlated with ASCVD independently of other cardiovascular risks, such as dyslipidemia, obesity, and atrial fibrillation (3, 6). Given that ASCVD is the leading cause of death worldwide (7), its early detection warrants investigation, especially in hyperthyroid patients.

The mechanism underlying ASCVD in Graves’ disease is related to the inflammatory effect of autoimmune disease (8), as well as the effect of excess thyroid hormone (9, 10). Such conditions will lead to vascular atherosclerosis (11, 12). Vascular atherosclerosis can be measured from adhesion molecules (13, 14)—such as intercellular adhesion molecule-1 (ICAM-1), vascular cell adhesion molecule-1 (VCAM-1), and E-selectin—and from arterial stiffness and thickness, which can be measured from pulse-wave velocity (PWV) and carotid intima media thickness (cIMT), respectively (15). Adhesion molecules are important proteins in endothelial activation, as they mediate the adhesion and migration of monocytes and macrophages in the endothelium, indicating a vascular response to inflammation (13, 14). Meanwhile, PWV and cIMT measurement show the structural and functional alteration of the vascular wall (16).

Considering its relation to ASCVD, the role of antithyroid drug treatment is becoming an interesting topic. Propylthiouracil (PTU) and methimazole are the most common antithyroid drugs prescribed by physicians (8). Bilir et al. (17) reported that PTU treatment for 18 months resulted in improved cIMT, whereas Chen et al. (18) found that PTU could inhibit the proliferation and migration of vascular smooth muscle cells (VSMCs). In contrast, we found only limited data about the effect of methimazole in atherogenesis. Napoli et al. (19) reported an improvement in flow-mediated dilation in Graves’ patients treated with methimazole (19, 20). However, data comparing the atherogenesis effect of PTU and methimazole in Graves’ disease are currently lacking. To understand the effects of the treatment for Graves’ hyperthyroidism on markers of vascular atherosclerosis, we examined newly diagnosed Graves’ disease patients and analyzed the level of adhesion molecules (ICAM-1, VCAM, E-selectin), PWV, and cIMT.

## Methods

### Study Design

This single-blind, randomized clinical trial study was conducted in Cipto Mangunkusumo General Hospital, Jakarta, Indonesia, from January 2019 to August 2020. Participants were randomly allocated to receive PTU or methimazole and followed up every month for 3 months. The study has been approved by the Ethical Committee of the Medical Faculty, Universitas Indonesia, Jakarta (ref KET-784/UN.2.F1/ETIK/PPM.00.02/2019). It has also been registered at clinicaltrials.gov (NCT05118542).

### Study Population

The participants in this study were all newly diagnosed Graves’ disease patients, aged 18–65 years, who had not undergone prior antithyroid drug treatment for more than 1 month. Graves’ disease was diagnosed from clinical manifestation of hyperthyroidism with diffuse goiter and then confirmed with TSH, fT4, and TSH receptor antibody (TRAb) measurement. Patients with pregnancy, a history of coronary heart disease, known malignancy, current use of immunosuppressive medication, sepsis, thyroid crisis, and allergic reaction or other severe side effects to antithyroid drugs were excluded from the study. All participants were informed and gave written informed consent prior to all trial-related activities.

### Data Collection

At the first visit, all eligible participants were invited to provide demographic data, including their age, sex, history of disease, history of psychological stress, and smoking history. Patients with prior antithyroid drug treatment were asked to stop the drug for 3 days to wash out the drug effects. The next day, participants were asked to fast overnight (10–12 h) and the venous blood was collected to measure thyroid function and adhesion molecules. Participants were also examined for carotid artery PWV and cIMT using ultrasonography examination. After finishing all examinations, participants were given PTU or methimazole according to their randomized allocation with dosage as instructed by their attending endocrinologist. All participants were followed up every month until the third month to evaluate the side effects, thyroid hormone levels, and markers of vascular atherosclerosis.

### Blood Collection

Peripheral blood was collected into no-anticoagulant vacutainers after overnight fasting. TSH, total T3, and fT4 levels were measured using electrochemiluminescence immunoassay (ECLIA) methods (Abbott, Abbott Park, Illinois, USA). TRAb was measured using ECLIA methods (Roche, Basel, Switzerland). Blood samples were sent to the university’s laboratory with a cold pack, then centrifuged at 3,000 rpm for 10 min to collect the serum, which was then stored at –80°C.

### Adhesion Molecules

Adhesion molecules (ICAM-1, VCAM-1, and E-selectin) were measured in serum samples using enzyme-linked immunosorbent assay methods (R&D Systems, Minneapolis, Minnesota, USA) according to the manufacturer’s protocol.

### PWV and cIMT

All ultrasonographic measurements were performed by an operator who was blinded to the intervention drugs. Doppler ultrasound examinations were conducted in the procedural room using the ultrasound linear vascular probe (4–13 MHz) as a tonometer and analyzed using quality arterial stiffness and quality intima-media thickness radiofrequency software (Esaote Medical Systems, Genova, Italy).

After the patient was placed in a supine position, the common carotid artery was shown in a longitudinal view. Anterior and posterior walls were visualized clearly. PWV and cIMT were automatically recorded. Each measurement was performed three times (with maximum differences of 15%) and then averaged for the final PWV and cIMT values. The left and right carotid arteries were measured individually because of anatomical differences (the left carotid artery is the main branch of the aorta, whereas the right carotid artery is a branch of the brachiocephalic artery).

### Statistical Analysis

Data were analyzed using IBM SPSS Statistics Version 23.0 (IBM, Armonk, New York, USA). Categorical data were presented in frequency (%) values, whereas numerical data were presented using the mean [standard deviation (SD)] if normally distributed or median [interquartile range (IQR)] if not normally distributed. To analyze the changes in parameters from baseline to the first- and third-month treatment, repeated analysis of variance (ANOVA) was performed. In addition, a general linear model test was performed to analyze the difference between the PTU and methimazole groups. For non-normally distributed data, transformation into normally distributed data was performed, and the data were presented as the geometric mean [95% confidence interval (CI)].

## Results

### Study Population

**Figure 1:**
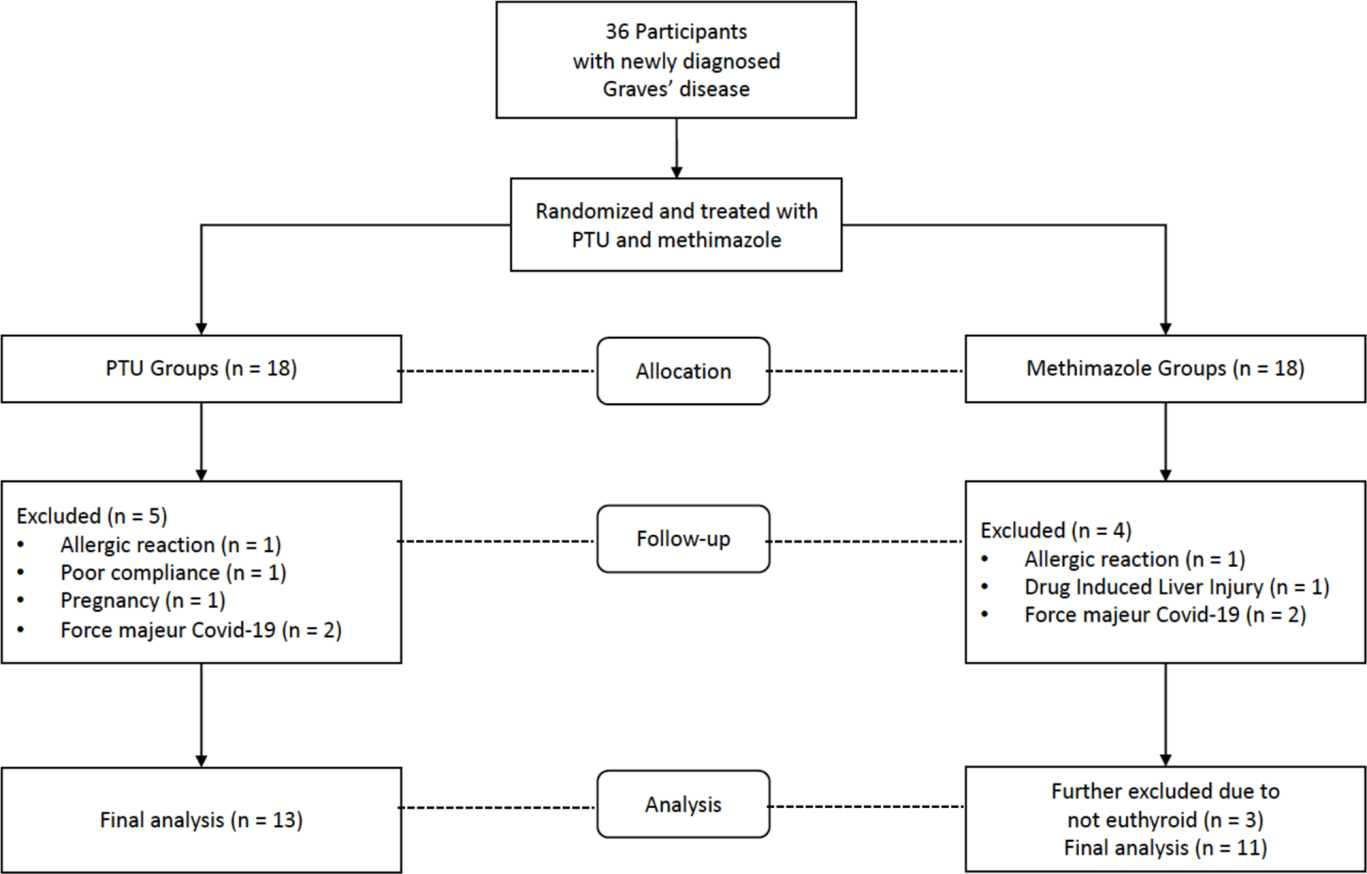
Consort flow diagram of study participants.

From July 2019 to June 2020, a total of 36 participants who fulfilled the inclusion criteria and were not disqualified as a result of the exclusion criteria were recruited for the study. Nine participants dropped out while under observation: Two participants experienced allergic reactions, one developed drug-induced liver injury, one became pregnant, one exhibited poor compliance to therapy, and four were lost to follow-up due to the coronavirus disease 2019 (COVID-19) pandemic. In addition, after 3 months of treatment, three participants did not reach a euthyroid state. Considering that the hyperthyroid state reflects ongoing inflammation, the final analysis was performed only on euthyroid participants (**Figure 1**).

### Baseline Characteristics

The baseline characteristics of the study participants are shown in **Table 1**. The mean ages of participants were 38.6 (SD 14) years in the PTU group and 41.1 (SD 9.5) years in the methimazole group. The female: male ratio in the PTU group was 11: 2, whereas it was 5: 6 in the methimazole group. There was a higher number of hypertensive patients in the PTU group [4 (30.8%) participants] compared with the methimazole group [1 (9.1%) participant)]. However, there were more patients with a smoking history in the methimazole group [8 (72.7%) participants] compared with the PTU group [2 (15.4%) participants].

**Table 1:** Baseline demographic characteristic, clinical symptoms, and physical examination of study participants.

Demographic characteristic, clinical symptoms, and physical examination	PTU (n = 13)	Methimazole (n = 11)
Age, mean (SD), years	38,6 (14)	40,1 (9,5)
Sex, n (%)		
Female	11 (84.6)	5 (45.4)
Male	2 (15.4)	6 (54.6)
History of hypertension, n (%)	4 (30.8)	1 (9.1)
Family history of thyroid disease, n (%)	4 (30.8)	5 (45.5)
Smoking history, n (%)	2 (15.4)	8 (72.7)
History of psychological stress, n (%)	3 (23.1)	3 (27.3)
Palpitation, n (%)	11 (85)	10 (91)
Frequent bowel movement or diarrhea, n (%)	5 (46)	7 (64)
Unintentional weight loss, n (%)	12 (92)	9 (82)
Dyspnea on effort, n (%)	4 (38)	5 (45)
Excessive sweating, n (%)	7 (61)	8 (73)
Heat intolerance, n (%)	7 (61)	7 (64)
Irritability, n (%)	2 (16)	4 (36)
Fatigue, n (%)	7 (61)	8 (73)
Warm moist hands, n (%)	5 (46)	3 (27)
Diplopia, n (%)	1 (8)	0 (0)
Systolic BP, mean (SD), mmHg	138 (11)	137 (14)
Diastolic BP, mean (SD), mmHg	92 (9)	96 (8)
Heart rate, mean (SD), x/m	107 (15)	108 (22)
Respiratory rate, median (IQR), x/m	18 (16-20)	18 (16-20)
Temperature, mean (SD), °C	36.9 (0.6)	36.7 (0.5)
Body weight, mean (SD), kg	57.9 (11.8)	61 (9.4)
Height, mean (SD), cm	161.5 (10.0)	163.9 (6.0)
Body Mass Index, median (IQR) kg/m^2^	22.2 (19.2–23.9)	24.0 (18.9–25.4)
Neck circumference, mean (SD), cm	33.3 (2.6)	34.2 (2.8)

BP, blood pressure; IQR, interquartile range; PTU, propylthiouracil; SD, standard deviation.

### Changes in Vascular Atherosclerosis Marker After 3 Months of Treatment

**Figure 2:**
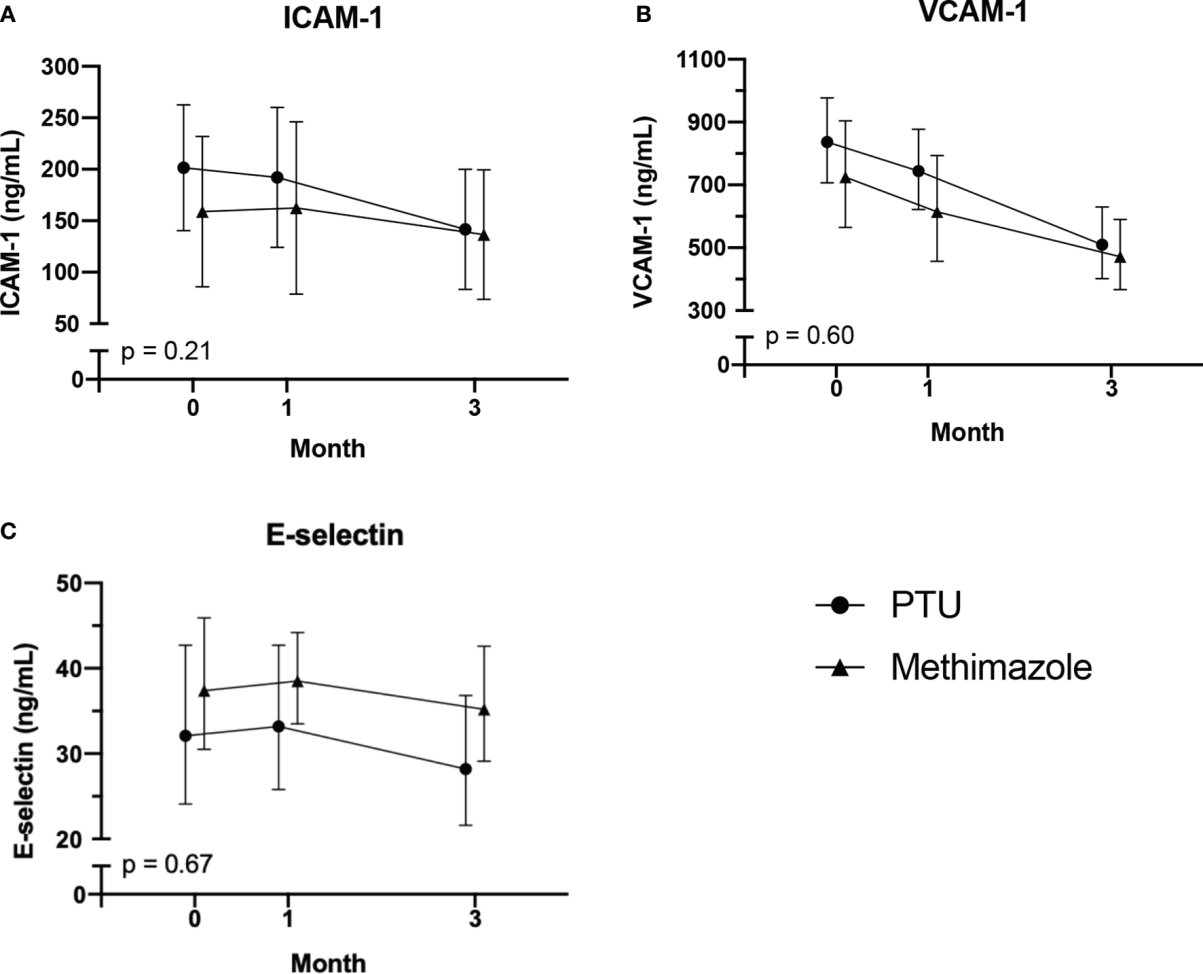
Changes in adhesion molecules: **(A)** ICAM-1; **(B)** VCAM-1; **(C)** E-selectin. After 3 months of antithyroid drug treatment. Intercellular adhesion molecule-1; VCAM-1 vascular cell adhesion molecule-1. The comparisons were assessed using the general linear model test.

**Figure 3:**
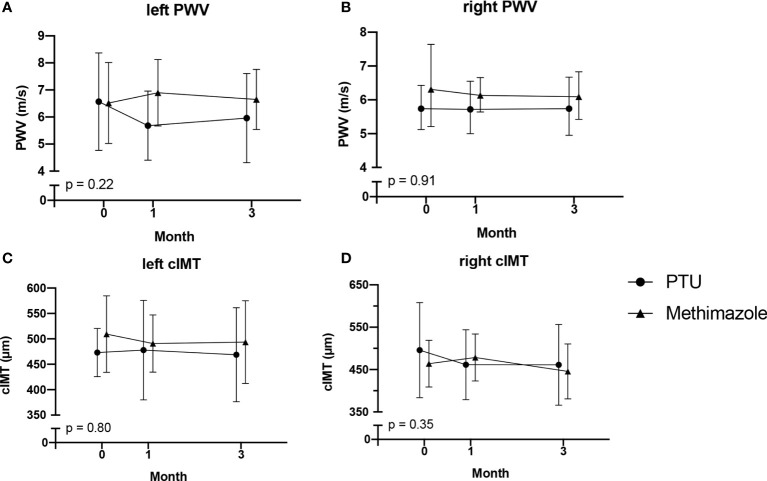
Changes in PWV: **(A)** left PWV; **(B)** right PWV; and cIMT: **(C)** left cIMT; **(D)** right cIMT. After 3 months of antithyroid drug treatment. PWV, pulse wave velocity; cIMT, carotid intima media thickness. The comparisons were assessed using the general linear model test.

After treatment for 3 months, significant improvements in ICAM-1, VCAM-1, and E-selectin levels were observed (**Table 2**). In the analysis of the treatment effects within each group, we found that in the PTU group, there were significant improvements in all ICAM-1 (p = 0.001), VCAM-1 (p < 0.001), and E-selectin (p = 0.045) parameters. Meanwhile, in the methimazole group, only improvement in the VCAM-1 level (p = 0.001) was observed (**Table 3**). However, in comparing the treatment effect between the two groups, we found no significant difference of the adhesion molecule improvement in the groups (**Figure 2**). In the analysis of the PWV and cIMT levels, we observed no significant changes after 3 months of antithyroid treatment, either between or within the groups (**Tables 2**, **3**, and **Figure 3**).

**Table 2:** Changes in vascular atherosclerosis marker after 3 months of anti-thyroid treatment.

Research Parameter	Baseline	After 1 Month of Treatment	After 3 Months of Treatment	p value
ICAM-1 (ng/mL)*^a^*	181.9 (68.9)	178.5 (75.5)	139.3 (59.3)	0.001*^c^*
VCAM-1 (ng/mL)*^b^*	777 (626–948)	657 (547–841)	445 (384–600)	0.001*^c^*
E-selectin (ng/mL)*^a^*	37.1 (14.7)	37.5 (12.6)	33.5 (12.8)	0.033*^c^*
Left PWV (m/s)*^a^*	6.55 (1.63)	6.24 (1.38)	6.28 (1.44)	0.59
Right PWV (m/s)*^b^*	5.99 (5.42–6.62)	5.90 (5.46–6.38)	5.90 (5.39–6.46)	0.91
Left cIMT (μm)*^b^*	482 (445.8–541.2)	480 (432.2–517)	482.5 (409.7–527.7)	0.58
Right cIMT (μm)*^a^*	481.2 (90.4)	469.4 (70.5)	454.1 (81.3)	0.29

Repeated ANOVA test.

a Data presented as mean (SD).

b Data presented as geometric mean (CI 95%).

c p-value < 0.05.

ICAM-1, intercellular adhesion molecule-1; VCAM-1, vascular cell adhesion molecule-1; PWV, pulse wave velocity; cIMT, carotid intima media thickness; SD, standard deviation; CI, confidence interval.

**Table 3:** Changes in vascular atherosclerosis marker after 3 months of anti-thyroid treatment in PTU and methimazole groups.

Research Parameter		PTU	Methimazole	p^β^ Value (Overall)
ICAM-1 (ng/mL)*^a^*	Baseline	201.4 (61.3)	158.8 (73.0)	0.21
	After 1 month of treatment	192.2 (68.1)	162.4 (83.8)	
	After 3 months of treatment	141.6 (58.4)	136.5 (63.0)	
	p^α^ value	0.001*^c^*	0.31	
VCAM-1 (ng/mL)*^b^*	Baseline	837 (707–977)	725 (565–904)	0.60
	After 1 month of treatment	744 (622–877)	614 (457–794)	
	After 3 months of treatment	510 (402–630)	472 (367–590)	
	p^α^ value	<0.001*^c^*	0.001*^c^*	
E-selectin (ng/mL)*^b^*	Baseline	32.1 (24.1–42.7)	37.4 (30.5–45.9)	0.67
	After 1 month of treatment	33.2 (25.8–42.7)	38.5 (33.5–44.2)	
	After 3 months of treatment	28.2 (21.6–36.8)	35.2 (29.1–42.6)	
	p^α^ value	0.045*^c^*	0.27	
Left PWV (m/s)*^a^*	Baseline	6.57 (1.80)	6.52 (1.50)	0.22
	After 1 month of treatment	5.68 (1.28)	6.90 (1.23)	
	After 3 months of treatment	5.96 (1.65)	6.65 (1.11)	
	p^α^ value	0.15	0.82	
Right PWV (m/s)*^b^*	Baseline	5.74 (5.12–6.43)	6.31 (5.21–7.64)	0.91
	After 1 month of treatment	5.72 (5.0–6.55)	6.13 (5.64–6.66)	
	After 3 months of treatment	5.74 (4.95–6.67)	6.09 (5.42–6.83)	
	p^α^ value	0.99	0.89	
Left cIMT (μm)*^b^*	Baseline	473.1 (47.4)	509.4 (75.4)	0.80
	After 1 month of treatment	477.8 (98.1)	490.8 (56.4)	
	After 3 months of treatment	468.9 (92.6)	493.6 (81.3)	
	p^α^ value	0.95	0.77	
Right cIMT (μm)*^a^*	Baseline	495.9 (112.3)	463.8 (55.3)	0.35
	After 1 month of treatment	461.5 (82.6)	478.6 (55.5)	
	After 3 months of treatment	461.2 (95.1)	445.7 (64.8)	
	p^α^ value	0.50	0.30	

p^α^ value for comparison before and after treatment. Repeated ANOVA test.

p^β^ value for comparison between PTU and methimazole groups. General linear model test.

a Data presented as mean (SD).

b Data presented as geometric mean (CI 95%).

c p-value < 0.05.

ICAM-1, intercellular adhesion molecule-1; VCAM-1, vascular cell adhesion molecule-1; PWV, pulse wave velocity; cIMT, carotid intima media thickness; SD, standard deviation; CI, confidence interval.

## Discussion

This study aimed to evaluate the differential effects of PTU and methimazole treatment in Graves’ disease on vascular atherosclerosis markers, presented by adhesion molecules, PWV and cIMT. After 3 months of treatment, not only a significant reduction in all adhesion molecules (ICAM-1, VCAM-1, and E-selectin) but also a better reduction from PTU groups were observed. However, we did not observe changes in the PWV or cIMT parameters after the interventions. Our results showed that in Graves’ disease, antithyroid drug treatment can result in a reduction of atherosclerosis risk. Moreover, in patients with high atherosclerosis risk, PTU may become the preferred antithyroid treatment.

Our study participants comprised of adult patients with newly diagnosed Graves’ disease. Similar to the study of Luigi et al. (21), the mean age of our participants was 38.6 years for the PTU group and 40.1 years for the methimazole group. In our study, more participants from the PTU group had hypertension, and more participants from the methimazole group had a history of smoking. Nakanishi, et al. (22) reported a 1.4 times increased risk of cardiovascular disease in hypertensive patients. Meanwhile, Aune et al. (23) reported a relative risk of 1.44 from patients with a history of smoking. Therefore, we assume that the risk of atherosclerosis in both groups from our study is relatively comparable.

Thyroid hormone has been linked to vascular atherosclerosis (1, 5, 24). Hypothyroidism is well understood to increase serum lipid levels—such as total cholesterol, LDL cholesterol, and apolipoprotein B—which are the main mechanism in the development of atherosclerosis plaque (5, 25), whereas recent studies also explore the role of hyperthyroidism in vascular atherosclerosis (3). Graves’ disease in particular, in addition to hyperthyroidism, also increases the risk of atherosclerosis because of its inflammatory property (4, 26). Our study has further explored the relationship between Graves’ disease and vascular atherosclerosis through adhesion molecules pathway. Adhesion molecules are proteins responsible for adhesion and recruitment of monocytes/macrophages into the vascular wall (27, 28). Increased levels of adhesion molecules will lead to adhesion and migration of monocytes into the intima media (29), resulting in the formation of foam cells and ultimately atherosclerosis plaque. These findings are in line with Bano et al. (3) reporting the arterial wall as a target of thyroid autoantibodies, thereby enhancing atherosclerosis plaque development. Consequently, it is important to maintain euthyroid state as both hypo- and hyperthyroidism may result in atherosclerosis formation.

In our study, we observed a reduction in ICAM-1, VCAM-1, and E-selectin levels after treatment with antithyroid drugs for 3 months. Wenisch et al. (30) and Jublanc et al. (31) reported higher levels of ICAM-1 and VCAM-1 in Graves’ disease compared with healthy control. Bilgir et al. (32) found elevated ICAM-1 and VCAM-1 levels even in subclinical hyperthyroidism patients, which then were reduced after reaching a euthyroid state. These findings indicate that elevated adhesion molecules caused by hyperthyroidism can be reduced by antithyroid drugs. Improvements in ICAM-1, VCAM-1, and

E-selectin could be attributed to the antioxidant effect of antithyroid drugs: As hyperthyroidism is controlled, inflammation is reduced (10), and therefore, the adhesion molecule expression will be reduced (31, 33, 34).

**Figure 4:**
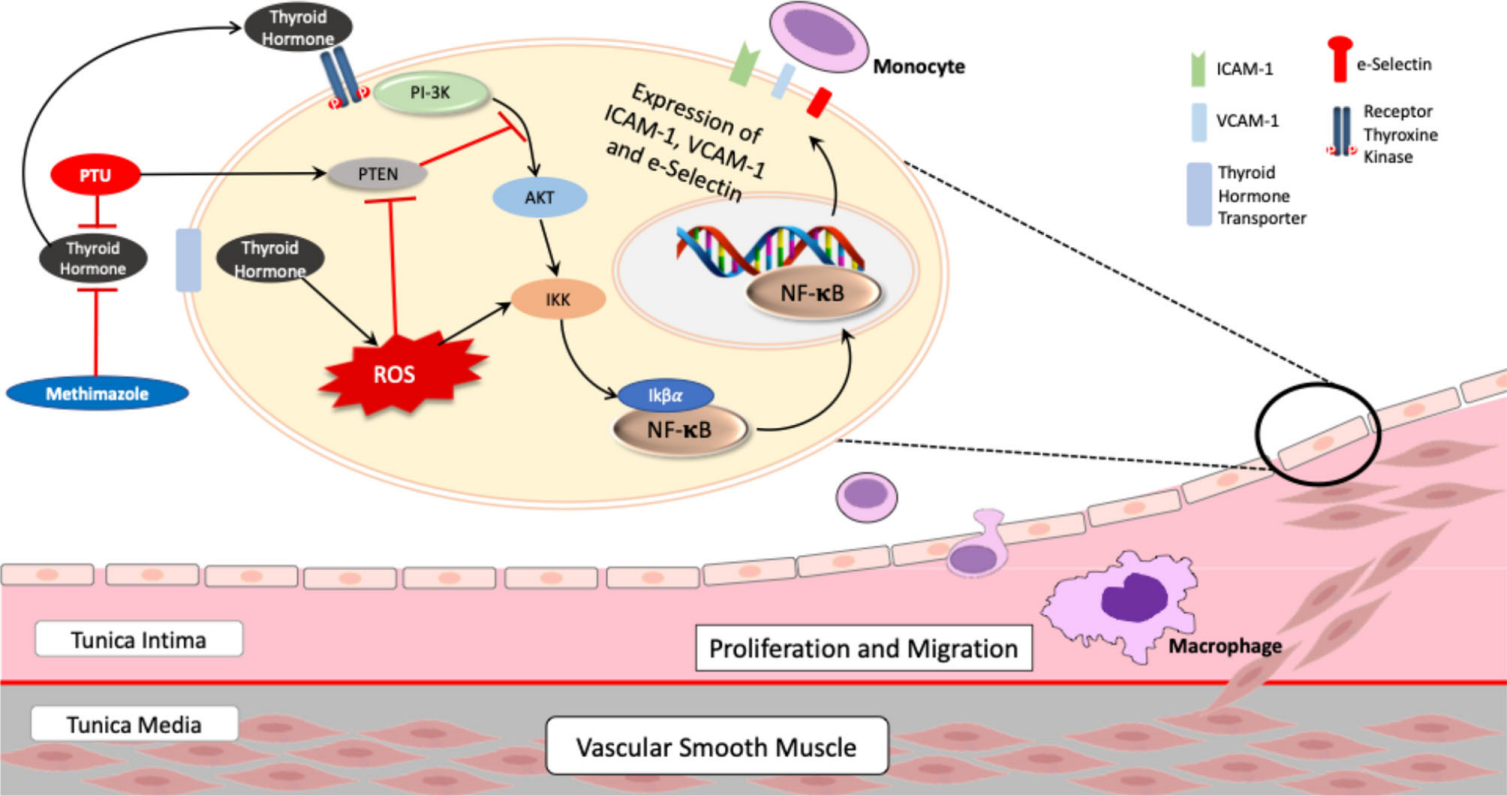
Proposed mechanism of PTU and methimazole in pathophysiology of atherosclerosis in Graves’ disease. AKT, protein kinase B; NF-κB, nuclear factor-kappa B; IKK, IKβ kinase; PI-3K, phosphoinositide 3-kinase; PTEN, phosphatase and tensin homolog; PTU, propylthiouracil; ROS, reactive oxygen species; intercellular adhesion molecule-1; VCAM-1, vascular cell adhesion molecule-1. Thyroid hormone induces activation of the PI-3K/AKT/IKK/NF-κB pathway, which in turn increase expression of adhesion molecules—ICAM-1, VCAM-1, and E-selectin. In addition, thyroid hormone also increases ROS, amplifying NF-κB activation through IKK. However, treatment with PTU can block the PI-3K/AKT/IKK/NF-κB pathway by inducing the expression of PTEN, a tumor-suppressor gene that can block the PI-3K/AKT pathway, resulting in less expression of adhesion molecules.

In the individual analysis, we observed that PTU could reduce all three adhesion molecules—ICAM-1, VCAM-1, and E-selectin—whereas methimazole only reduced VCAM-1. However, we did not find a statistically significant difference in our comparison between the two groups. Therefore, although we could not conclude that PTU is superior to methimazole, we suggested that PTU might have a better mechanism for improving endothelial dysfunction. In their study, Chen et al. (18) reported that an additional effect of PTU, other than its antithyroid effect, was to increase the expression of the phosphatase and tensin homolog (PTEN) gene. PTEN is an enzyme known as a tumor-suppressor gene (35, 36), and it can inhibit phosphatidylinositol-kinase (PI-3K) activity (37). *Via* the PI-3K/AKT pathway (29, 38, 39), PI-3K activates IKβ kinase (IKK); this results in the activation of IKβα, which can bind to nuclear factor-κB (NF-κB) (40) and increase the expression of ICAM-1, VCAM-1, and E-selectin (38). Therefore, PTU can reduce adhesion molecules levels by increasing the PTEN expression. Meanwhile, since data and an understanding of the basic mechanism of methimazole are still lacking, we suggest that methimazole reduces adhesion molecules mainly *via* its antithyroid activity (20, 41, 42). In accordance with these findings, we can propose a mechanism that explains how PTU might have a better mechanism in improving endothelial dysfunction (**Figure 4**).

Arterial stiffness and thickness are surrogate markers for endothelial function that can be used to predict atherosclerosis (43, 44). However, our study could not conclude that there is a relationship between hyperthyroidism and PWV or cIMT. Baseline data for PWV and cIMT levels of our participants were within the normal range. The normal range for PWV in adults aged 20–60 years is 5.86–8.15 m/s (45), whereas it is 400–600 μm for cIMT (17, 45, 46). These findings were different from other studies reporting that hyperthyroidism is related to an increase in PWV or cIMT (17, 47, 48). In addition, we did not find significant changes in PWV or cIMT after antithyroid treatment in our study. These findings were different from a study by Bilir et al. (17) reporting a significant reduction of ICAM-1 levels after 12 months of treatment with PTU, as well as a study by Inaba et al. (49) reporting a reduction in arterial stiffness 3 months after normalization of thyroid hormone levels. These differences may have been caused by the short-term onset of Graves’ disease in our participants, which in turn resulted in normal levels of PWV and cIMT at the baseline.

To our knowledge, our study was the first to explore the differential effect of both PTU and methimazole treatment on vascular atherosclerosis markers in Graves’ disease. Our findings of the early reduction of adhesion molecules, as well as the potential benefit of PTU compared with methimazole, were the strong points of the study. However, our study has several limitations. The dropout number was high because of drug reactions and the COVID-19 pandemic. In addition to that, further attempts to continue the study were proven unsuccessful, as new participants from February 2020 until August 2020 were unwilling to join the study because of the COVID-19 pandemic. Consequently, the study power was reduced. The follow-up duration of the study was 3 months, intended to reach a euthyroid state so that early changes in vascular atherosclerosis can be observed; therefore, a long-term effect especially on PWV or cIMT could not yet been found significant.

## Conclusions

In conclusion, while we observed improved adhesion molecules after antithyroid treatment in Graves’ disease, no significant changes were observed in PWV or cIMT. Moreover, PTU may have a better mechanism for preventing atherosclerosis compared with methimazole. Further study that continues to the stage of disease remission is warranted to explore the effect of hyperthyroidism in Graves’ disease on atherosclerosis and the potential benefit of PTU compared with methimazole in more depth.

## Data Availability Statement

The raw data supporting the conclusions of this article will be made available by the authors, without undue reservation.

## Ethics Statement

The studies involving human participants were reviewed and approved by the Ethical Committee of the Faculty of Medicine Universitas Indonesia. The patients/participants provided their written informed consent to participate in this study.

## Author Contributions

WW and IS contributed to the conception, design, and acquisition; critically drafted the manuscript; revised the manuscript; gave the final approval; and agreed to be accountable for all aspects of work ensuring integrity and accuracy. IA and NN contributed to the conception, design, and acquisition; critically revised the manuscript; and gave the final approval. All other authors contributed to the article, revised the manuscript, and approved the submitted version.

## Funding

This research was supported by PUPTN Kemenristekdikti of Republic Indonesia Research Grant with reference number NKB-2752/UN.2.RST/HKP.05.00/2020.

## Conflict of Interest

The authors declare that the research was conducted in the absence of any commercial or financial relationships that could be construed as a potential conflict of interest.

## Publisher’s Note

All claims expressed in this article are solely those of the authors and do not necessarily represent those of their affiliated organizations, or those of the publisher, the editors and the reviewers. Any product that may be evaluated in this article, or claim that may be made by its manufacturer, is not guaranteed or endorsed by the publisher.
